# Cardiovascular Involvement in Sjögren’s Syndrome

**DOI:** 10.3389/fimmu.2022.879516

**Published:** 2022-05-06

**Authors:** Fabiola Atzeni, Francesco Gozza, Giacomo Cafaro, Carlo Perricone, Elena Bartoloni

**Affiliations:** ^1^ Rheumatology Unit, Department of Clinical and Experimental Medicine, University of Messina, Messina, Italy; ^2^ Rheumatology Unit, Department of Medicine and Surgery, University of Perugia, Perugia, Italy

**Keywords:** risk factors, drugs, heart, Sjögren’s syndrome, cardiovascular risk

## Abstract

Sjögren Syndrome (SS) seems to be associated with a greater “overall risk” of cardiovascular (CV) and cerebrovascular events. Although not conventionally considered a feature of the disease, CV events represent a major burden in SS patients. CV risk is the consequence of a complex combination of multiple factors, including traditional risk factors and disease-related mechanisms. A complex relationships between disease-related features, endothelial dysfunction and traditional risk factor has been suggested. Several drugs are available for treating the systemic manifestations of SS, however they have shown positive effects on different outcomes of the disease, but until today the data on the role of these drugs on CV events are scarse. Given these data, the aim of this review was to evaluate the risk of CV risk in primary SS and the effect of the drugs on this manifestation.

## Introduction

Sjögren’s Syndrome (SS) is one of the most frequent rheumatic diseases, with a prevalence of 0.1-0.6% in the adult population, a male-female ratio of approximately 1:9 and an average age at diagnosis of 50 years (1). It is characterized by a lymphocytic infiltrate in the lacrimal and salivary glands causing keratoconjunctivitis sicca and xerostomia, which are the main clinical manifestations of the disease (2). Other clinical manifestations are vaginal dryness, non-productive cough, salivary gland swelling and systemic symptoms (arthralgia, fatigue and general discomfort) (3, 4).

SS can be primary (pSS) or associated to other connective tissue disorders, in particular Systemic Lupus Erythematosus (SLE) and rheumatoid arthritis (RA). Extra glandular involvement may be present in about one third of patients with pSS. The most affected organs are thyroid, lungs, gastrointestinal tract, blood, kidneys, skin and central and peripheral nervous system. The involvement of the cardio-vascular system is less commonly observed in these patients. Severe complications as acute pericarditis and myocarditis have been rarely reported (5), although the best-known cardiac complication is congenital heart block associated with positive anti-Ro/SSA antibodies, which is one of the manifestations of neonatal lupus. However, the latter is rare in adults and is not consistently correlated with the presence of anti-SSA/Ro antibodies (6). Moreover, patients with SS secondary to anti-phospholipid antibody syndrome (APS) are characterized by an increased risk of ischemic stroke.

Numerous studies have highlighted how acute systemic inflammation and chronic vasculitis are known to be associated with endothelial dysfunction (7). Furthermore, in some rheumatic diseases including SLE and RA, the tendency to develop atherosclerosis is one of the major causes of mortality (8).

Primary SS appears to be associated with a greater “overall risk” of CV and cerebrovascular events. However, evidence related to prevalence of cerebrovascular events appears conflicting. In fact, pSS can be associated with vasculitis, which may cause an increased risk of haemorrhagic strokes due to the inflammatory infiltrate in the vascular wall (9). However, other studies have reported either a greater risk of ischemic cerebrovascular events (10) or have shown no difference between patients with pSS and the control group (11).

The purpose of this review was to evaluate the risk of CV risk in pSS and the effect of medications on this manifestation.

## Cardiovascular Events in pSS: What Is the Evidence?

Although not conventionally considered a feature of the disease, CV events represent a major burden in pSS patients, similarly to what has been extensively described in other autoimmune diseases such as RA and SLE.

Several studies demonstrated an increased risk of CV and cerebrovascular events in pSS patients, compared to the general population. The main limitation to the interpretation of these data is the wide heterogeneity of outcomes and of variable definitions. In fact, the definition of cardio and cerebro-vascular events, beyond stroke and acute myocardial infarction (AMI), may include or exclude transient ischemic attacks (TIA), arrhythmias, angina, valvular disease and thromboembolic events.

However, the prevalence of CV involvement in a very wide sense was demonstrated to be about 61.6% in pSS subjects compared to 29.7% in healthy controls (HC) (12). More specifically, in a cohort study, cerebrovascular and CV events had a significantly higher prevalence in pSS compared to HC (2.5% vs 1.4% and 1.0% vs 0.4%, respectively) (13). The hazard ratio of pulmonary embolism (PE), deep vein thrombosis (DVT) and venous thromboembolism (VTE) was demonstrated to be 4.07, 2.80 and 2.92, respectively, compared to healthy subjects, especially in the first years after diagnosis (14). These data have not been confirmed in all studies published on the topic (15), which may be due to differences in terms of ethnicity and selection of study participants.

Nonetheless, a recent meta-analysis including 9 studies, confirmed a higher risk of CV (OR=1.30, 1.09 to 1.55) but not cerebrovascular events (OR=1.31, 0.96 to 1.79). Similarly, a more recent meta-analysis including 14 studies reproduced similar results and found a higher rate of coronary disease (RR 1.34, 1.06 to 1.38), cerebrovascular disease (RR 1.46, 1.43 to 1.49), heart failure (RR 2.54, 1.30 to 4.97) and thromboembolism (RR 1.78, 1.41 to 2.25) in pSS patients. However, the higher rate of CV events was not associated with a significant increased risk of mortality (16).

The most likely reason underlying the consistency but not universal agreement of the results regarding the higher frequency of major CV events in pSS is likely mostly attributable to their time-dependency. In fact, risk factors usually require a long exposure time before actually causing the outcome. As a consequence, a very long observation time is required to detect the impact of one single risk factor, as the disease itself, especially when multiple other and more frequent factors, as other CV risk factors, affect the outcome.

In order to analyse the effect of risk factors earlier enough, surrogate outcome measures are commonly employed. A surrogate outcome measure is a measurable variable that is more sensitive to change than major outcomes but also directly associated to them. When these conditions are satisfied, a surrogate marker can partially replace the measure of the main outcome.

In the field of CV research, multiple surrogate outcome measures have been validated and demonstrated to be associated to pSS. Similarly to RA, pSS patients show a higher carotid intima-media thickness (a surrogate marker of atherosclerosis) than HC, and having pSS is an independent risk factor for arterial wall thickening (17). Another surrogate of accelerated atherosclerosis is arterial stiffness, measured as pulse-wave velocity, which has been found to be higher in pSS compared to HC (18–21). Other studies also demonstrated a lower ankle-brachial index (ABI) in pSS patients (22) and a subclinical involvement of the heart. In fact, higher prevalence of valvular regurgitation, pericardial effusion, pulmonary hypertension, increased left ventricular mass, systolic dysfunction and lower coronary reserve were demonstrated in pSS patients (18, 23, 24). Some Authors suggest that the higher prevalence of heart failure may be a consequence of ventriculo-arterial coupling, i.e. the premature return of the pulse wave caused by reduced arterial elastance may contribute to left ventricular hypertrophy and, in turn, diastolic dysfunction (24).

Although there is no doubt that pSS patients are burdened by a higher CV risk, the causes and factors accounting for it are yet to be fully understood. The most supported theory is that CV risk is the consequence of a complex combination of multiple factors, including traditional risk factors and disease-related mechanisms. The full comprehension of the single determinants is mandatory in order to be able to properly assess and address them from a clinical point of view, thus offering the best care to patients.

## Traditional Risk Factors

The first obvious consideration to make is whether pSS patients have a different prevalence of traditional CV risk factors compared to the general population, which may account for the excess CV morbidity. Indeed, multiple observational studies demonstrated a higher prevalence of hypertension in pSS (28-50% vs 11.3-25.6% in the general population) (13, 25, 26). However, other studies show opposite results (27), mostly due to differences in methodological approaches and definition of hypertension. Interestingly, one study found that hypertension tends to be under-treated in pSS patients compared to the general population (25).

Lipid metabolism seems also to be altered in pSS patients. Several studies found a higher prevalence of dyslipidaemia in pSS patients as compared to the general population (21-22.5% vs 4.2-9.5%) (13, 25, 26, 28, 29). However, findings are very heterogeneous when a more detailed analysis is carried out. In fact, some studies found higher levels of triglycerides, others lower high-density lipoprotein (HDL) cholesterol levels but not total cholesterol, others a higher rate of statin use as an indirect measure of dyslipidaemia. Additionally, a significant minority of reports showed apparently contrasting results (28). To further support the hypothesis of an altered lipid metabolism, higher levels of the adipokines – cytokines secreted by adipose tissue – resistin and adiponectin have been reported (26).

Data on metabolic syndrome, obesity and diabetes are even more conflicting and scarce. The prevalence of metabolic syndrome and diabetes seems to be higher in pSS (39.4% vs 16.9% and 27% vs 13%, respectively) (26, 27), which is in contrast with other studies reporting a lower prevalence of obesity (11% vs 21%) and diabetes mellitus (4% vs 7%) (13). As previously mentioned, these differences can globally be explained by different methodology in terms of patients enrolment and variable definitions. In contrast, it is now well established that smoking is significantly less common among pSS patients compared to the general population but also to patients with other autoimmune diseases, probably due to the worsening of sicca symptoms induced by cigarette smoke (30, 31). However, the paucity of data on this topic significantly hamper a full understanding of the picture.

## Disease-Related Factors

Albeit SS patients seem to have an overall higher prevalence of traditional CV risk factors, this is not sufficient to account for the whole excess CV morbidity. Few studies investigated CV events in distinct subset of SS patients, thus data so far are limited and mostly derive from indirect evidence. However, some reports suggest that the role of anti-SSA/Ro and anti-SSB/La is significant to the point that the rate of cerebral infarction and VTE may be higher compared to the general population (HR = 1.7, 1.0 to 2.9 and 3.1, 1.9 to 4.8, respectively) only in the subgroup of seropositive subjects (32). Although these data need to be confirmed, there is little doubt that the presence of autoantibodies has an impact of CV morbidity. In fact, through a neural network analysis of a large cohort of SS patients, the presence of anti-SSA/Ro antibodies turned out to represent a hub, connecting the two main pillars of CV disease phenotypes in SS, i.e. ischemic (such as myocardial o cerebral infarction) and non-ischemic events (such as heart failure) (33, 34). To further support a close link between disease features and CV events, the same study showed that ischemic events were mostly associated to variables characterizing extra-glandular involvement (vasculitis, low complement, leukopenia, cryoglobulins) while non-ischemic events were mainly linked to the presence of sicca syndrome (34). Additionally, a higher prevalence of anti-phospholipid antibodies was found in SS patients, potentially contributing to CV disease (4).

## The Network Model

In a complex disease such as SS, characterized by a wide spectrum of alterations of immunity and inflammatory processes, it is intuitive to think that a strict and compartmentalized model of CV risk into traditional and disease-related risk factors cannot reliably represent reality.

Consequently, it is important to investigate the complex relationships between disease-related features, endothelial dysfunction and traditional risk factor. In line with the findings described above, a reduction of ABI is associated to seropositivity for anti-Ro/SSA and anti-La/SSB (22) and a higher prevalence of CV events is associated to central nervous system (CNS) involvement and immunosuppressive treatment (a surrogate marker of severe disease) (13). As endothelial dysfunction is an early marker of atherosclerosis - an inflammatory process involving vessel walls - an association between metabolic syndrome, dyslipidaemia and serum concentration of interleukin (IL)-1β and IL-6 in SS patients is not surprising (26). Additionally, endothelial dysfunction seems more pronounced in patients with joint involvement, parotid enlargement (35) and, more generally, with active disease measured by European League Against Rheumatism (EULAR) Sjogren’s syndrome disease activity index (ESSDAI) (36, 37).

However, contrasting data have been published too, finding increased aortic stiffness in patients with no circulating anti-Ro/SSA antibodies (21) and an association between traditional risk factors and the presence of extra-glandular disease features (27). Other studies, instead, were not able to find any significant associations between endothelial dysfunction and disease features (38). Indeed, endothelial dysfunction is one of the earliest changes that characterize atherosclerosis. Its main features are reduced vasodilatory response, alterations in the expression of surface adhesion molecules and deficient repair mechanisms and endothelial cell turnover. All these features lead to accelerated atherosclerosis. Multiple studies have investigated endothelial changes in SS. In terms of functional assays, SS patients display a deficient flow-mediated dilation (FMD) (38, 39) and nitrate-mediated vasodilation (NMD) (35). These functional aspects are in agreement with higher levels of circulating asymmetric dimethylarginine (ADMA), responsible for reduced production of nitric oxide in SS compared to HC (18, 39).

As far as endothelial damage repair mechanisms are concerned, SS patients have an increased number of circulating endothelial microparticles (EMP), endothelial progenitor cells (EPC) (40) and angiostatin (39). Very interestingly, EPCs, involved in endothelial repair, seem to be higher in early disease, while EMPs, a marker of damage, in late disease, which leads to the hypothesis that repair mechanisms remain functional in early disease and wane at later stages, potentially contributing to accelerated atherosclerosis (40). Additionally, a recently described T cell population involved in the coordination of endothelium repair mechanisms, thus named angiogenic T cell (Tang), is expanded in peripheral blood and salivary glands of SS patients and may play a role in the induction or persistence of endothelial dysfunction (36).

It is therefore clear that to fully understand the determinants of CV risk in SS, we cannot disregard the intricate connections among the multiple players involved. Due to the unavoidable influence they exert on each other, dissecting the role of each factor independently is unreasonable. Thus, a multifaceted approach to the topic is the only realistic path to further extend our knowledge in this complex field, ultimately to improve patient care.

## Mechanisms of Atherosclerosis

**Figure 1:**
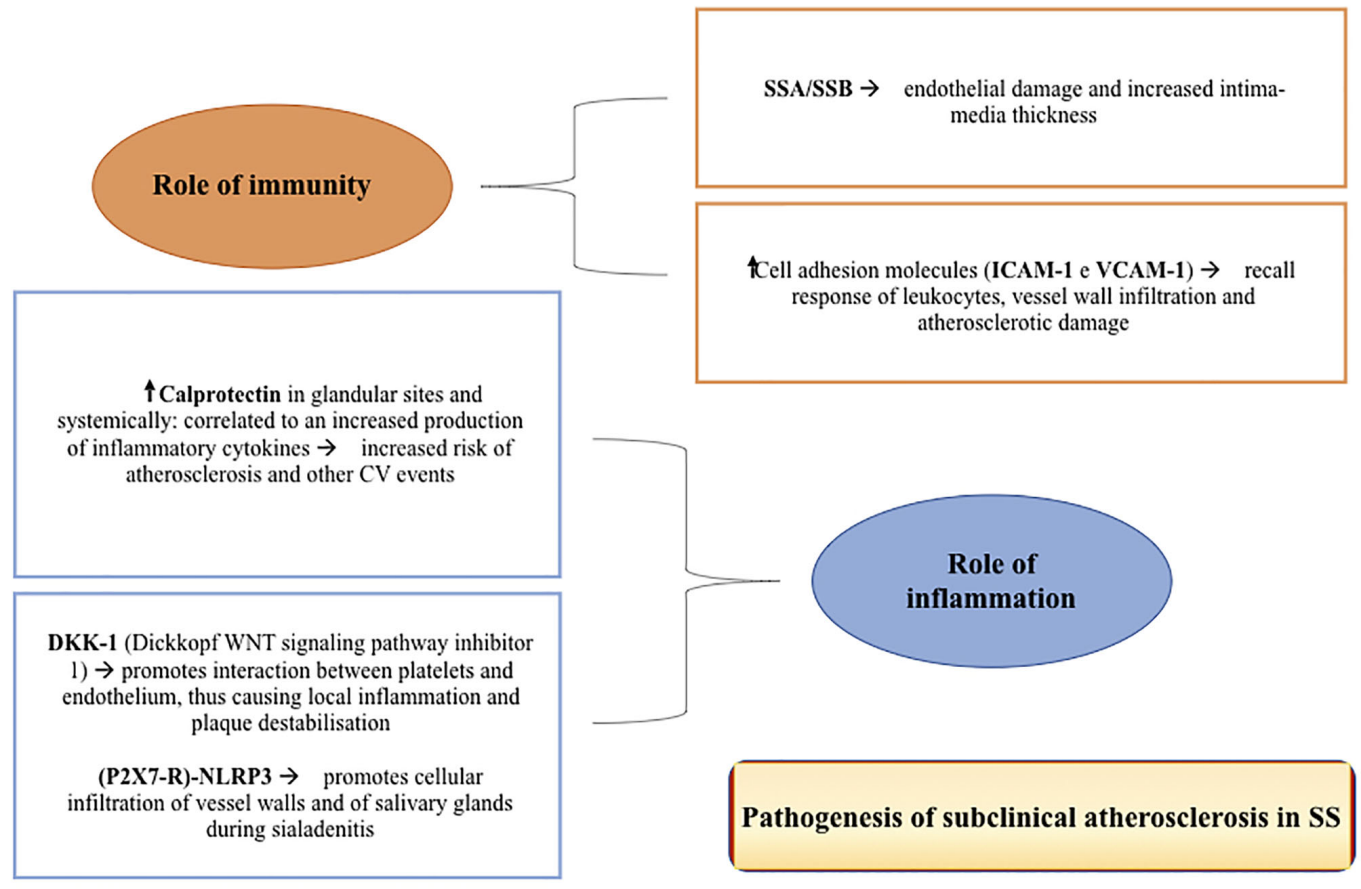
The figure shows some of the main pathogenic mechanisms of subclinical atherosclerosis in Sjogren’s Syndrome.

Atherosclerosis is a multifactorial disorder that tends to begin early in life, but with late-onset clinical manifestations. The isolation of immune cells including lymphocytes and macrophages from atherosclerotic lesions indicates an involvement of the immune system in the etiopathogenesis of this disorder (41, 42) (**Figure 1**). Inflammation may play a crucial role in the exacerbation of atherosclerosis. Systemic autoimmune rheumatic diseases (AIRDs) lead to an increased risk of morbidity and mortality due to cardiovascular events, especially following atherosclerotic events. This could be attributed to the traditional risk factors for atherosclerosis, and to the treatment with specific drugs such as corticosteroids (43, 44).

The infiltration of atherosclerotic plaques by immune cells, as well as their activation, can occur as a result of numerous trigger factors, such as microbial infections (45). It is thought that these cells have a fundamental role in promoting the progression of atherosclerosis and this is based on several studies. For instance, in C57BL/6 mice the depletion of CD4 + and CD8 + T lymphocytes may lead to a net decrease in the formation of lipid striae. Furthermore, mice with SCID (severe combined immunodeficiency) and apolipoprotein E (ApoE) knockout mice, showed a reduction in aortic lipid striae in 73% of cases, compared to mice with a normal immune system. Finally, the transfer of CD4 + T lymphocytes to immunodeficient mice led to an increase in the dimensions of atherosclerotic lesions (46). Therefore, it is clear that atherosclerosis has an autoimmune setting, with immunity cells releasing numerous cytokines within the plaques [interleukin (IL), tumour necrosis factor (TNF)-alpha, and Platelet-Derived Growth Factor (PDGF)].

During the atherosclerotic process, immune cells may respond specifically to certain molecules, including oxidized low-density lipoproteins (OxLDLs), heat-shock proteins (HSP), and Beta2 glycoprotein-I (β2GPI) (41). This is shown by an increase of these molecules in the atherosclerotic plaques (47). On the other hand, some autoantibodies have been associated with a greater risk of atherosclerosis, as evidenced in some animal studies carried out with anti-cardiolipin antibodies (48). OxLDLs, once phagocytised by macrophages, cause the transformation of macrophages into foam cells, in turn promoting the progression of atherosclerosis. Anti-OxLDL antibodies are present in both healthy patients and patients with AIRDs (49), but in the latter, especially in patients with SLE, SSc and systemic vasculitis, these antibodies appear to be present at higher levels than controls (49–51). A correlation between anti-OxLDL antibody levels and total immunoglobulin levels was also observed in SLE patients (50).

## Role of Immunity

The immune system dysregulation plays an important role in the pathogenesis of early atherosclerosis in patients with pSS. The positivity of anti-SSA/Ro and -SSB/La antibodies, two well-known markers of the disease and generally associated with systemic manifestations (35, 52), may correlate with endothelial dysfunction and with an increase in the intima-media thickness. The latter, along with alteration in the NO-induced vasodilatation, may be associated with and predictive for leukopenia, marker of high disease activity and higher risk of CV events (13, 35, 52). The organic and functional damage of the smooth muscle cells in the vessel tunica media may be determined by cellular infiltration of the sub-endothelial space. This is demonstrated by the higher levels of ICAM-1 and VCAM-1 adhesion molecules found in patients with SS. These molecules may recruit leukocytes with consequent infiltration of the vascular wall and induction of atherosclerotic damage (35).

## Role of Inflammation

The real contribution of inflammation in the pathogenesis of atherosclerotic damage in SS is not fully understood. Although two studies showed a correlation between C-reactive protein (CRP) levels and the risk of developing atherosclerosis and CV events (18, 25, 27), most studies found no differences in CRP levels in patients with SS compared to HC (17, 27, 35, 38, 53–55). An important role is played by calprotectin (53), a protein expressed in the cytoplasm of neutrophils and an excellent indicator of innate immune activity. This protein is highly expressed in patients with SS in both glandular sites, correlating with the Focus Score (FS) and the circulation, being associated with the increased production of cytokines such as IL-1β, IL-6, TNF-α, IFN-γ, IL-10 and IL-22. This molecule seems to represent an important prognostic factor for CV events, regardless of other risk factors (56). Another noteworthy mechanism is the Wingless-type (Wnt) signaling pathway, which plays a key role in the process of inflammation regulation (17). In fact, there is a correlation between the DKK-1 molecule (Dickkopf WNT signalling pathway inhibitor 1) and atherosclerosis. This molecule appears to be more expressed in endothelial cells and atherosclerotic plaques, where it promotes the interaction between platelets and the endothelium, in turn causing local inflammation and plaque destabilization (57). Recent studies on the (P2X7R)-NLRP3 complex involved in the pathogenesis of sialadenitis in SS showed a close correlation between inflammation and infiltration of the glands and those observed in the vessel wall in atherosclerosis (58).

## Therapeutic Approaches in Atherosclerosis

Therapeutic strategies for atherosclerosis and for any other CV disease should be evaluated on the basis of the specific clinical manifestations (59). In patients at increased risk of thrombosis and vascular occlusion, such as in SLE and APS, antiaggregating therapy (aspirin) should be recommended (60). The key point of the treatment is prevention (61). The increased risk of developing atherosclerosis or other CV events in patients suffering from autoimmune diseases is caused not only by the autoimmune state itself, but also by the complications of these diseases and by the treatments carried out to treat them (for example corticosteroids) (62). Careful monitoring of blood pressure, periodic checks of laboratory parameters and lipids, regular exercise and the use of drugs, such as statins and folic acid, if necessary, are some of the main therapeutic strategies to prevent the formation or evolution of these pathological conditions (63). Further approaches are still under study, such as immunomodulation (immunosuppressive drugs, administration of IV immunoglobulins (IVIg), induction of tolerance with autoantigens) (64), use of inhibitory cytokines, gene therapy and bone marrow transplantation.

### Traditional Cardiovascular Risk Factors and Therapy

Regarding the correlation between these CV risk factors and the clinical and laboratory manifestations of SS, it was found that these risk factors are more present in subjects suffering from more severe and widespread forms of the disease, including subjects with pulmonary or neurological manifestations (13). In fact, it seems that the risk factors themselves contribute to the progression of inflammation, resulting in greater involvement of the main internal organs. Furthermore, patients with more severe forms of the disease and in turn with an increased need of treatments with glucocorticoids (GCs) and immunosuppressive drugs, have a greater risk of developing CV risk factors. According to some studies, on the other hand, hydroxychloroquine (HCQ) appears to play a protective role on the development of risk factors for CV events in the aforementioned population (27).

## Treatment of Sjögren’s Syndrome

The management of pSS has not undergone significant changes in the last few decades and continues to rely on the symptomatic treatment of sicca syndrome and on the use of a variety of immunosuppressive drugs to treat its systemic manifestations (65). Although the great amount of drugs used to treat the systemic manifestations of the disease, data on CV manifestations are scarce and extrapolated according to their mechanisms of action, as summarized in **Table 1**.

**Table 1:** The table highlights the efficacy of the drugs most used in the treatment of SS, particularly on eye dryness, dry mouth, systemic manifestations (fatigue, arthralgia/arthritis, pain, and other organ manifestations) and on the main CV manifestations (atherosclerosis and other conditions).

Drugs		Mechanism of action	Effects on eye dryness	Effects on xerophthalmia	Effects on systemic manifestations	Effects on CV manifestations
**Topical Drugs**						
**Ciclosporin A**		Inhibition of T lymphocytes proliferation and down-regulation of proinflammatory cytokines	–			
**Systemic Drugs**						
**Pilocarpine, Cevimeline (**		Parasympathomimetic drugs	–	–		
**GC**		Anti-inflammatory and immunosuppressive drugs. Upregulation of anti-inflammatory genes and suppression of the production of proinflammatory transcription factors. Th1 response Inhibition and production of numerous cytokines.	°	°	-	*
**HCQ**		Antimalarial drug with immunomodulatory activity.	°	°	–	–
**MTX**		Immunosuppressive drug, folic acid antagonist.	-	-	-	
**CSA**		Inhibition of T lymphocytes proliferation and down-regulation of proinflammatory cytokines.	°		–	
**MMF**			°	°	-	
**Belimumab**		Monoclonal antibody anti-BAFF.	–	–	–	
**RTX**		Anti-CD20 chimeric recombinant monoclonal antibody.	-	°	°	
**IGU(+ HCQ e GC)**		Small molecules inhibiting B lymphocytes function	–	–	–	
**Key**						
–	Prevalence of beneficial effects					
°	Conflicting results on the effects of the drug					
*	Prevalence of negative effects					

CsA, ciclosporin A; RTX, rituximab; HCQ, Hydroxychloroquine; GCs, glucocorticoids; MMF, mycophenolate mofetil; MTX, methotrexate; IGU, iguratimod.

### Therapy for the Clinical Manifestations of Sicca Syndrome

Muscarinic receptor agonists (e.g., pilocarpine, cevimeline) have shown to improve xerostomia and xerophthalmia in several randomized clinical trials (RCTs) (66). Topical cyclosporine has been approved for the treatment of xerophthalmia when the use of artificial tears is insufficient (67, 68).

### Topical Cyclosporine A

Xerophthalmia is one of the main manifestations of pSS. However, it can also occur in patients not affected by pSS (69). In the first case, the lymphocytic infiltrate of the lacrimal glands and conjunctiva can trigger an inflammatory process that causes the loss of muciparous goblet cells. This leads to the development of dry eye, which is responsible for ocular discomfort and visual disturbances (70). It can also severely limit daily activities, reduce quality of life and even lead to depression in cases of moderate/severe disease (69). Inflammation of the ocular surface plays a crucial role in the development and evolution of xerophthalmia. Numerous studies have shown how the levels of inflammatory cytokines (IL-17, TNF-α, IL-6, metalloproteinases and chemokines) are increased in both pSS and associated SS (71–79). Therefore, anti-inflammatory drugs are an important part of the treatment of the dry eye syndrome.

Ciclosporin A (CsA) is an immunomodulating and anti-inflammatory drug used topically with few side effects, which are instead frequently observed in patients treated with CCs. Its mechanism of action consists in the inhibition of T-cell proliferation and the downregulation of inflammatory cytokines in the lacrimal gland and conjunctiva (80, 81). Treatment with topical CsA (0.05%) showed a significant improvement in the Schirmer’s Test (ST) after about twelve months of therapy in patients with xerostomia secondary to SS, and after about 6 months in patients with xerostomia not associated with SS. An improvement was also observed in terms of tear breakup time (TBUT), but this was not statistically significant in SS patients. A significant decrease was observed in the OSDI (Ocular staining disease index) score for both groups of patients (affected and not affected by SS) (82). Topical CsA may lead to an improvement in ocular symptoms by reducing surface inflammation, in turn causing an increase in the density of conjunctival goblet cells and an improvement in lacrimation induced by sensory stimulation (83). In fact, sensory nerve fibers depart from the ocular surface and along their path connect with efferent fibers of the autonomic nervous system. Inflammation of the ocular surface, by reducing corneal sensitivity, causes a reduction in the response of the lacrimal glands to ocular damage (83). Finally, an improvement in the Corneal Staining Score (CSS) was also shown (82, 84–87). However, the results obtained showed a greater efficacy of topical treatment with CsA in subjects suffering from dry eye not related to SS, probably because in patients with SS there is also a systemic inflammatory process.

## Systemic Therapy

Several studies focused on conventional systemic immunosuppressive therapy (prednisone, cyclosporin, azathioprine, methotrexate) and other drugs (dehydroepiandrosterone, nizatidine, rebamipide), have not demonstrated efficacy in the control of syndrome sicca, although mild benefits have been reported for some drugs (1, 66, 88–90). Furthermore, conventional and biological DMARDs, widely used in other rheumatological diseases, did not show significant clinical effects in pSS. Therefore, their use remains small and limited to some specific patient subsets (91).

### Glucocorticoids

To date, the therapeutic landscape of SS is poor and based on the symptomatic treatment of glandular manifestations and on the use of immunosuppressive and/or anti-inflammatory drugs to treat systemic manifestations (92–97). Moreover, the use of corticosteroids in this disease has not been extensively studied yet, but it is based on the results obtained in other rheumatic diseases, such as SLE and RA (98, 99).

Glucocorticoids (GCs) are steroid hormones produced by the zona fasciculata of the adrenal cortex and their secretion is regulated by the hypothalamus-pituitary-adrenal axis, with circadian rhythms. GCs derive from a single precursor, cholesterol, due to the activation of various enzymes, similarly to aldosterone and dehydro-epi-androsterone (DHEA). GCs are involved in different physiological and metabolic processes, such as water and electrolyte balance, immunity, growth, cardio-vascular function, reproduction, and cognitive function (100).

CBG (corticosteroid-binding globulin) is the main serum protein with the function of transporting GCs. These drugs, once bonded to this protein, are kept in an inactive form. A small part of CBGs is instead free in the plasma (not bonded to proteins), and this represents its active form. Two different enzymes (11β-hydroxysteroid dehydrogenase type 1 and 2) help maintaining the balance between the active and inactive form of GCs, and they are controlled by various factors including cytokines and interleukins (TNF and IL-1β) (101). The active form is lipophilic, and it is therefore able to pass through the cell membrane and penetrate inside the cell, producing genomic (slow) and non-genomic (rapid) effects.

The receptors for GCs (GRs) are found inside the cell and regulate the expression of genes responsive to GCs positively (transactivation) or negatively (trans-repression) through different mechanisms (100). One of these is binding to the GREs (glucocorticoid response elements), which are palindromic nucleotide sequences of the promoter of the target gene. Alternatively, GRs can bind and activate additional proteins such as transcription factors (STAT3, STAT5 NF-kB), in order to enhance the activation or repression of some genes. Finally, they can act with a combined mechanism that involves binding to DNA and the activation of specific proteins (102, 103).

In clinical practice, synthetic GCs are used. They are structurally similar but not identical to endogenous cortisol. These show different characteristics (pharmacokinetics, bioavailability, cross-reactivity with mineralocorticoids) and have different specificity and potency. Their use, since the discovery of these drugs, has spread enormously in the field of inflammatory and autoimmune diseases such as RA, SLE, rheumatic polymyalgia, bronchial asthma, COPD, chronic inflammatory bowel diseases (IBD), immune-mediated glomerulonephritis, multiple sclerosis and numerous other conditions (104) including SS.

We do not have large-scale studies to evaluate the real benefits of GCs therapy in patients with SS. It has been observed that they reduce glandular swelling, but the results on the sicca syndrome are conflicting. In a small, randomized trial, neither treatment with prednisone (30 mg/die) nor with piroxicam (20 mg/die) compared with placebo, showed statistically significant results after 6 months on both lacrimal and salivary glands functionality and on salivary gland infiltration (105). GCs are sometimes used as drugs for systemic manifestations, as in many other rheumatic diseases, and this was demonstrated in an analysis involving 1120 Spanish patients with pSS, in whom low doses of GCs (the equivalent of prednisone 20 mg/die) were used in 19% of patients (106). Furthermore, in an Italian multicentric study, the use of GCs was reported in 45% of 1343 patients with pSS (13), while in the GEAS-SS cohort involving 1580 patients (107) it was reported in 85% of patients (excluding those with lymphoma who received corticosteroid therapy in combination with RTX). To date, the risk-benefit ratio of these drugs in pSS is not yet completely clear, and the idea still exists that these drugs should be prescribed at the lowest dosage and for the shortest possible time to control the disease and avoid the development of CV events, given their well-known adverse effects (108).

## Conventional Immunosuppressive Drugs and Synthetic DMARDs

### Hydroxychloroquine

Hydroxychloroquine (HCQ) is a drug approved several years ago by the Food and Drug Administration (FDA) for the prevention and treatment of malaria. HCQ has also shown to be very effective in reducing disease flares and in controlling signs and symptoms of SLE, dyslipidaemia, platelet aggregation, etc. (109). It is effective in subacute cutaneous lupus (annular erythema) (110), a skin manifestation also described in SS. Data on HCQ in SS is scarce. Some observational studies and some open-label trials have shown an improvement in arthralgia and myalgia in patients with increased indices of inflammation and hypergammaglobulinemia (111). By contrast, a randomized placebo-controlled trial showed no improvement in dryness, fatigue and pain after 6 months of therapy. The long-term efficacy that HCQ showed in SLE in both reducing the risk of systemic complications of lymphoma and determining an increase in survival (112), has yet to be demonstrated in pSS. Since this drug is generally manageable, well tolerated and quite safe, it is currently one of the first-choice drugs for the treatment of pSS and may likely play a role in the prevention of CV risk.

However, it has been observed that at high doses in the treatment of some oral diseases, HCQ can lead to the onset of lethal arrhythmias related to a QT prolongation, such as the torsade de pointes arrhythmia (113–115). Therefore, it would be advisable to evaluate the association of hydroxychloroquine with other drugs responsible for QT prolongation.

### Methotrexate

A one-year pilot study of methotrexate (MTX) (0.2 mg/kg per week) in patients with. pSS showed improvement in xerostomia and xerophthalmia, arthralgia/arthritis, frequency of parotid swelling and purpura (116). However, no improvement in salivary and lacrimal gland function indices was observed and no data is available on CV involvement (116).

### Azathioprine

In a randomized study that included 25 patients with pSS, low-dose azathioprine (AZA) therapy (1 mg/kg/die) showed no effect on disease activity over a 6-month period (117). However, AZA is commonly used for the treatment of some extra-glandular manifestations, such as interstitial pneumonia, chronic autoimmune hepatitis and myelopathy (118).

### Leflunomide

In an open-label pilot study, leflunomide (LEF) (20 mg/die) showed modest benefit in 15 patients with active pSS (119). However, in a study involving RA patients, LEF was associated with a worsening of xerostomia in a group of 45 patients with RA and associated SS, compared to a group of 30 patients with pSS (120).

### Mycophenolate Mofetil

In an open-label pilot study in 11 patients with pSS, mycophenolate mofetil at doses up to 1440 mg/die for 6 months did not result in a statistically significant improvement in oral and ocular dryness. However, therapy was associated with a significant reduction in hypergammaglobulinemia and rheumatoid factor (RF) and an increase in complement levels and white blood cell counts (121).

### Cyclosporin

In a randomized controlled trial, cyclosporine therapy (5 mg/kg/die) showed an improvement of xerostomia compared to placebo after 6 months of therapy, although it did not demonstrate any benefit on ocular symptoms and scores for assessment of eye and oral dryness. In another open-label phase II study, treatment with cyclosporine (2 mg/kg/die) showed efficacy in reducing joint swelling and stiffness and in improving DAS28 after 16 weeks of therapy (122, 123).

## Biological Disease-Modifying Anti-Rheumatic Drugs (DMARDs)

Some clinical studies have been carried out using TNF-α (infliximab and etanercept) and interleukin 1 (IL-1) (anakinra) as targets, but with poor results (124–126). A recent study evaluated the efficacy of tocilizumab (anti-IL-6) in patients with SS, but it did not show an improvement neither in sicca symptoms nor in the systemic manifestations of the disease compared to placebo (127). Similarly, abatacept, a drug acting as inhibitor of CD80/CD86-CD28 co-stimulation, has not shown positive results, despite some previous studies reported an improvement in disease activity (assessed by ESSDAI) and a reduction in cytokine and autoantibody levels (128, 129).

### Belimumab

Belimumab is a fully human IgG1 monoclonal antibody directed against the cytokine BAFF (B-cell activating factor), also known as BlyS (B lymphocyte stimulator). In consideration of the high affinity towards BAFF, belimumab can bind both its forms, the soluble and the cell membrane bound. In this way, it inhibits the binding of BAFF with its three receptors: BAFF-R (or TNFR-13C), BCMA (B-cell maturation antigen or TNFR-17), and TACI (transmembrane activator and CAML interactor or TNFR-13B) (130). BAFF-R can only bind the BAFF ligand, while the other two also act as a receptor for the “sister” cytokine APRIL (a proliferation-inducing ligand).

BAFF is synthesized by immune cells, including macrophages, neutrophils, dendritic cells, B lymphocytes, activated T lymphocytes, NK cells activated by IL-2, and stromal cells. BAFF is initially expressed as a cell membrane surface protein and can be processed into its soluble form by the furin protease. In order to bind and activate BAFF-R, BAFF needs to form homotrimers or heterotrimers composed of BAFF and APRIL. Once activated, BAFF-R is crucial for the survival and maturation of naïve and transitional B cells.

BCMA is responsible for the survival of plasma cells and TACI mediates the B-independent T-cellular response to certain antigens, the regulation of the B-cell compartment and the “immunoglobulin class switching”.

Therefore, the therapeutic use of belimumab interferes with the homeostasis of mature B lymphocytes, immature B lymphocytes and plasma cells, while it has no action on the progenitors of B lymphocytes and memory B lymphocytes. Given its mechanism of action, belimumab has been used to treat systemic autoimmune diseases, including ANCA-associated vasculitis, LES, RA, myasthenia gravis and SS (130, 131).

The efficacy of belimumab was evaluated in an open-label study (BELISS) conducted for 28 weeks, followed by an extension trial for a further 24 weeks. The primary endpoint (improvement of two of the five predefined items: dryness, fatigue, musculoskeletal pain, systemic disease activity, and biomarkers of B cell activity), was achieved in 18/31 patients during the first 28 weeks and maintained in 13/15 patients during the following 24 weeks. The improvement in dryness, fatigue and pain symptoms was visible as early as week 28. The systemic disease activity showed improvement starting from week 52, while salivary flow and the Schirmer’s test did not show any changes. The quality of life, set as a secondary endpoint and assessed by the Short Form 36 (SF-36), showed an improvement in the physical component starting from week 52, although it did not show statistically significant results in the first 28 weeks (132, 133). Further double-blind studies are needed to confirm these promising results.

### Rituximab

Rituximab (RTX) is a chimeric recombinant monoclonal antibody directed against the CD-20 antigen. This antigen is an integral membrane protein expressed by most B cells (normal and malignant) and responsible for their activation, proliferation and differentiation (134). It is absent only in pro-B cells and plasma cells (135).

RTX consists of a murine antigen-binding domain connected with the constant region of human immunoglobulin G1 (IgG1). Once connected to CD-20, RTX causes a spatial reorganization of the CD-20 molecules within the phospholipid bilayer of the cell membrane, and in turn depletion of them through mechanisms of complement-dependent cytotoxicity (CDC), antibody-dependent cytotoxicity (ADCC) and phagocytosis by the activated reticuloendothelial system. These mechanisms ultimately lead to the apoptosis of B cells (136).

Since the discovery of the presence of autoantibodies, hypergammaglobulinemia and an increased risk of developing B-cell lymphoma, RTX has been one of the most studied drugs for the treatment of SS. However, studies carried out so far have not shown statistically significant clinical efficacy in the enrolled cohorts of patients.

#### Efficacy of RTX in Exocrine Gland Function and Sicca Syndrome

The functionality of the salivary glands is generally assessed through the following indices: unstimulated whole saliva (UWS) stimulated whole saliva (SWS) and VAS for xerostomia. Numerous studies have shown non-significant changes in salivary flow after RTX therapy (137–140), while a double-blind, randomized, placebo-controlled study showed a 24-week benefit of overall salivary flow and visual analogical score (VAS) pain (141). The TRACTISS study found that UWS values were maintained in subjects treated with RTX, while a worsening of this parameter was seen in patients treated with placebo.

A recent study evaluated histologic samples of salivary glands, highlighting a reduction in glandular inflammation and a decrease of lymphoepithelial duct damage. In another clinical trial, RTX was demonstrated to reduce the B cells infiltrate and promote the structural repair of the salivary glands (142, 143). The TRACTISS study reported a statistically significant improvement in salivary gland ultrasound score after RTX treatment in comparison to placebo-treated control group (144). Schirmer’s test results differ markedly across studies (137, 141). Other parameters, such as TBUT, Van Bijsterveld score and Rose Bengal test, showed significant changes in lacrimal gland function after RTX therapy. By contrast, in other studies, a modest, not statistically significant change in salivary gland function was reported (138–140).

#### Efficacy in Systemic Manifestations

The efficacy of RTX on systemic manifestations is not fully understood. Two RCTs demonstrated the ineffectiveness of RTX therapy in the treatment of pSS systemic manifestations evaluated by ESSDAI score (137, 145). By contrast, a prospective cohort study showed an improvement of ESSDAI score in 28 patients with pSS (146). An improvement was also observed in the SF-36 score (138).

#### Efficacy on Laboratory Parameters

Numerous studies have reported the efficacy of RTX in reducing the levels of FR and IgM (137, 138, 141, 147). B cell depletion has been reported in some trials (138). Conversely, the role of RTX in reducing anti-SSA/SSB antibody levels is questionable (138, 140).

#### Efficacy in the Treatment of Non-Hodgkin’s Lymphoma (NHL)

Despite the contribution of anti-CD20 monoclonal antibodies in increasing survival in different types of B-cell lymphoma, the eradication of the disease remains a challenge. In fact, in most cases, patients experience relapses (148).

### Combination Therapies

One of the most important challenges in the treatment of pSS to date, is the lack of therapeutic strategies to prevent lymphoma in high-risk patients, and once arisen, of valid therapeutic alternatives to deal with it. The combination of RTX with belimumab has given encouraging results in the treatment of SS-related B-cell lymphoproliferative MALT disorders with BAFF over-expression in comparison to monotherapy without reporting major adverse events (149).

## Other Therapies

### Iguratimod

Iguratimod (IGU) is a small-molecule drug widely used in China and Japan for the treatment of various rheumatic diseases (150). This drug inhibits the function of B cells, reducing the production of immunoglobulins and various inflammatory cytokines, including IL-1, IL-6, IL-8 and TNF (151). Moreover, numerous studies have reported a therapeutic efficacy of IGU against pulmonary fibrosis and osteoporosis (152). A recent meta-analysis demonstrated that IGU therapy was associated with an improvement of lacrimal and salivary gland function when used in combination with HCQ and GCs (153). The superiority of the treatment with IGU may be determined by the regulation of some B-lymphocyte subpopulations, the reduction in the production of immunoglobulins and the inhibition of pro-inflammatory T cells proliferation. Furthermore, IGU may reduce BAFF levels and the proportion of plasma cells, thus leading to a decrease in the production of IgG and in turn to a reduction in antibody-mediated damage.

Moreover, IGU determined an improvement in inflammatory parameters, IgG and RF levels, platelet count and disease indices (ESSDAI and ESSPRI) when associated with HCQ and GCs, without an increased risk of developing adverse events (153). Hence, it may also have protective effects on CV involvement. However, further studies are needed to evaluate the actual efficacy and safety of this drug.

### Future Perspectives: Selective Small Molecules

#### Type I Interferons

The role of interferon (IFN) in SS is well known. A study found that 2/3 of pSS patients had elevated IFN levels associated with hypergammaglobulinemia, antibody positivity (ANA and anti-SS-A/Ro antibodies) and high FS (154). Therefore, IFN may represent an important therapeutic target for the treatment of the disease.

A phase II study evaluated the efficacy of a monoclonal antibody (BIIB059) directed against the blood dendritic cell antigen 2 (BDCA2) on dendritic cells (the major producers of IFN) in patients with active cutaneous lupus erythematosus. Preliminary results have proved to be promising. Since IFN represents a common element in SLE and SS, it is believed that this drug can also be used in SS (155). In an ongoing RCT, the efficacy of tofacitinib, a Janus Kinase inhibitor, is being evaluated in patients with SS. This drug may be able to block the production of type I and II IFN (156). Ustekinumab, an anti-IL-12p40 antibody that blocks IL-12 and IL-23, thus inhibiting the IFN pathway, is being evaluated in an open-label phase I study (157). Fingolimod (158), a drug used in multiple sclerosis (MS) interfering with the sphingosine-1-phosphate (S1P) pathway, could also be used in SS, as well as its derivatives (e.g., ozanimod) (159), since the S1P receptor is also expressed in the salivary and lacrimal glands. Inhibitors of mTOR and AKT, drugs that interfere with the activation of T cells and with the regulation of the cell survival program, and other numerous molecules (BTK, Syk, Tyk) are being studied in order to evaluate their possible role in SS (160–165).

## Conclusions

In conclusion, surely pSS patients should receive a complete diagnosis, aimed at determining the extent and severity of the disease, the subset of the disease and the impact of the fatigue. The therapeutic approach is substantially similar in case of pSS or associated SS. Recent published recommendations include non-pharmacological and preventive interventions above all, such as hygiene measures, abstention from cigarette smoking, and counseling on a correct diet and appropriate use of drugs, etc. In addition, artificial tears and artificial saliva can be used, and if symptoms do not resolve, some patients may benefit from secretagogue drugs (pilocarpine and cimeveline) (166). In patients with moderate/severe involvement, a number of immunosuppressive drugs (synthetic and biological DMARDs) can be used. RTX should be used in case of severe organ manifestations not responsive to other treatments, such as cryoglobulinemic vasculitis. Treatment of fatigue primarily involves a low-impact aerobic exercise program. In patients with depression, anxiety or attention deficit, tests must be carried out in order to identify those who will need specific therapies. The treatment of the main cardiopulmonary manifestations (interstitial pneumonia, pulmonary hypertension and myocarditis) is based on the use of high-dose GCs often in combination with MMF or cyclophosphamide (CYC), or even RTX can be considered. Finally, the treatment of lymphoma in patients with SS should be carried out following the same protocols used for patients without SS. However, although most of the drugs used in the treatment of Sjogren’s syndrome have shown positive effects on different outcomes of the disease, to the best of our knowledge, we have poor data about the role of these drugs on CV events and surely further studies are needed to investigate the effect of these therapies on CV outcome in pSS patients.

## Author Contributions

FA, FG, GC, EB, and CP designed and performed the literature search. FA and EB drafted the narrative review and edited the manuscript. All authors read the manuscript, revised it for intellectual content, approved the final version, and agree to be accountable for all aspects of the work.

## Conflict of Interest

The authors declare that the research was conducted in the absence of any commercial or financial relationships that could be construed as a potential conflict of interest.

## Publisher’s Note

All claims expressed in this article are solely those of the authors and do not necessarily represent those of their affiliated organizations, or those of the publisher, the editors and the reviewers. Any product that may be evaluated in this article, or claim that may be made by its manufacturer, is not guaranteed or endorsed by the publisher.
